# Epigallocatechin-3-Gallate Modulates Postoperative Pain by Regulating Biochemical and Molecular Pathways

**DOI:** 10.3390/ijms22136879

**Published:** 2021-06-26

**Authors:** Rosalba Siracusa, Francesco Monaco, Ramona D’Amico, Tiziana Genovese, Marika Cordaro, Livia Interdonato, Enrico Gugliandolo, Alessio Filippo Peritore, Rosalia Crupi, Salvatore Cuzzocrea, Daniela Impellizzeri, Roberta Fusco, Rosanna Di Paola

**Affiliations:** 1 Department of Chemical, Biological, Pharmaceutical and Environmental Sciences, University of Messina, 98168 Messina, Italy; rsiracusa@unime.it (R.S.); rdamico@unime.it (R.D.); tgenovese@unime.it (T.G.); livia.interdonato@yahoo.it (L.I.); aperitore@unime.it (A.F.P.); salvator@unime.it (S.C.); rfusco@unime.it (R.F.); dipaolar@unime.it (R.D.P.); 2 Department of Biomedical, Dental and Morphological and Functional Imaging, University of Messina, 98125 Messina, Italy; fmonaco@unime.it (F.M.); cordarom@unime.it (M.C.); 3 Department of Veterinary Sciences, University of Messina, 98168 Messina, Italy; egugliandolo@unime.it (E.G.); rcrupi@unime.it (R.C.)

**Keywords:** pain, epigallocat-echin-3-gallate, rat

## Abstract

Treating postoperative (PO) pain is a clinical challenge. Inadequate PO pain management can lead to worse outcomes, for example chronic post-surgical pain. Therefore, acquiring new information on the PO pain mechanism would increase the therapeutic options available. In this paper, we evaluated the role of a natural substance, epigallocatechin-3-gallate (EGCG), on pain and neuroinflammation induced by a surgical procedure in an animal model of PO pain. We performed an incision of the hind paw and EGCG was administered for five days. Mechanical allodynia, thermal hyperalgesia, and motor dysfunction were assessed 24 h, and three and five days after surgery. At the same time points, animals were sacrificed, and sera and lumbar spinal cord tissues were harvested for molecular analysis. EGCG administration significantly alleviated hyperalgesia and allodynia, and reduced motor disfunction. From the molecular point of view, EGCG reduced the activation of the WNT pathway, reducing WNT3a, cysteine-rich domain frizzled (FZ)1 and FZ8 expressions, and both cytosolic and nuclear β-catenin expression, and the noncanonical β-catenin–independent signaling pathways, reducing the activation of the NMDA receptor subtype NR2B (pNR2B), pPKC and cAMP response element-binding protein (pCREB) expressions at all time points. Additionally, EGCG reduced spinal astrocytes and microglia activation, cytokines overexpression and nuclear factor kappa-light-chain-enhancer of activated B cells (NFkB) pathway, downregulating inducible nitric oxide synthase (iNOS) activation, cyclooxygenase 2 (COX-2) expression, and prostaglandin E2 (PGE2) levels. Thus, EGCG administration managing the WNT/β-catenin signaling pathways modulates PO pain related neurochemical and inflammatory alterations.

## 1. Introduction

In recent years, the undertreatment of acute pain was identified as a major health-care concern. Clinical analysis showed that only the 25% of surgical patients had satisfactory relief of acute pain [1]. Despite significant improvements in pain management, researchers suggest that postoperative (PO) pain is still inadequately managed [2,3]. The lack of a clinically meaningful progress in the PO pain analgesia is probably due to the limited efficacy and side effects of the available analgesic drugs [4,5]. Treatment of PO pain continues to be an important clinical goal. It is of fundamental importance to discover the mechanisms underlying PO pain, as well as to find medications to treat it. Despite numerous implicated processes and decades of investigation, the molecular and cellular mechanisms underlying PO pain remain uncertain, and therapeutic approaches for managing PO pain are limited. It has been described that central neuronal sensitization is involved in PO pain and hyperalgesia [6,7]. In vivo neurological studies have displayed increased rates and prevalence of spontaneous activity of spinal neurons in the dorsal horn after surgery [8,9]. However, the complete role of central sensitization in the pathophysiology of persistent PO pain is elusive [9]. A rat model for PO pain has been characterized by Brennan et al. [10]. In particular, the model consists of a surgical incision in the plantar hind paw and is characterized by enhanced responsiveness to mechanical stimuli [11]. At the end of the surgery, after the recovery from anesthesia, the allodynia and the reduced withdrawal thresholds are maxima and remains significant for several days before progressively decreasing [12]. Thus, investigating the role of the spinal activation in the perception and persistence of PO pain is an important challenge. It has been reported that the neuronal alterations that occur in neuropathic and bone cancer pain involve the activation of the WNT signaling [13]. 

WNTs are a family of proteins serving as long- or short-range signaling mediators in the management of cellular processes, such as differentiation, proliferation, and migration, during the development of the cardiac and central nervous system [14,15]. In humans, 19 members of these secreted lipid-modified signalling proteins have been identified [16]. In particular, WNT pathways include noncanonical β-catenin–independent signaling pathways and canonical β-catenin signaling pathways [14,17,18,19,20]. In the canonical pathway, WNT ligands link to the cysteine-rich domain frizzled (FZ) receptors which, in turn, activate several intracellular signaling cascades, including pro-inflammatory events. β-Catenin is a multi-purpose protein that interrelates with transcription factors to induce target gene transcription. The noncanonical β-catenin–independent signaling pathways consist in the release of intracellular calcium and the activation of the subsequent intracellular pathways [19,21]. Therefore, we imagined that targeting the WNT pathways with natural small molecules without side effects that would interfere with the analgesic drugs may be an effective strategy to counteract PO pain. 

Green tea has anti-inflammatory, anti-oxidant, anti-diabetic, and hypolipidemic effects. It increases insulin glucose uptake and insulin activity and reduces glucose absorption [22]. Moreover, green tea has a positive outcome in cases of cardiovascular diseases, cancer, oxidative stress, neurological diseases, infections, and hypercholesterolemia [23,24,25,26,27]. One of the most abundant active substances (up to 63%) in green tea is the epigallocatechin-3-gallate (EGCG) [28]. It has important activities, such as anti-oxidant, hypolipidemic, anti-inflammatory, anti-obesity, anticancer, and antidiabetic [29,30]. It shows an inhibitory effect on the WNT/β-catenin signaling pathways in human osteoarthritis chondrocytes [31]. The aim of this study was to evaluate the effect of the EGCG on the perception and persistence at different days of PO pain through the modulation of the canonical and noncanonical β-catenin-independent WNT signaling pathways, neurochemical and inflammatory modifications.

## 2. Results

### 2.1. Effect of EGCG Administration on Mechanical Allodynia, Thermal Hyperalgesia, and Motor Dysfunction Induced by PO Pain

**Figure 1:**
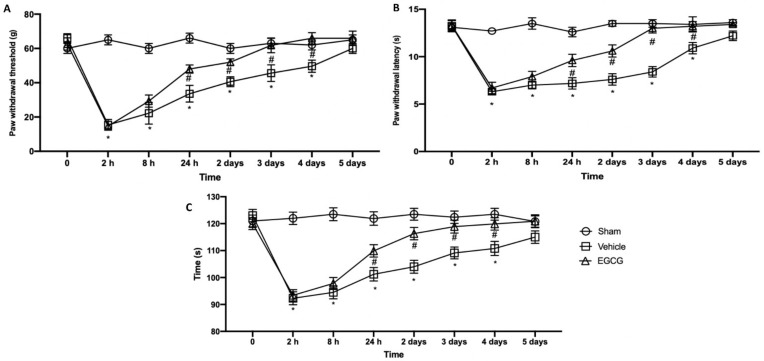
EGCG administration reduced mechanical allodynia, thermal hyperalgesia and motor dysfunction: von Frey test (**A**), plantar test (**B**) and rotarod test (**C**). A *p*-value of less than 0.05 was considered significant. * *p* < 0.05 vs. sham, # *p* < 0.05 vs. vehicle.

**Figure 2:**
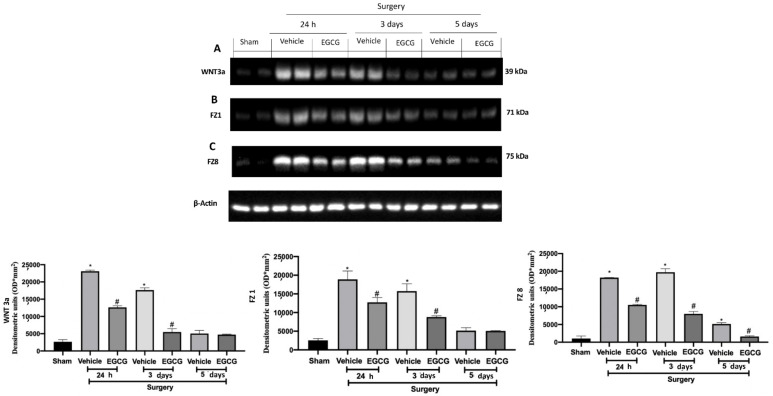
EGCG administration reduced WNT/FZ/β-catenin pathway activation: Western blot analysis of: WNT3a (**A**), FZ1 (**B**), FZ8 (**C**) expression. A *p*-value of less than 0.05 was considered significant. * *p* < 0.05 vs. sham, # *p* < 0.05 vs. vehicle.

Behavioral analyses were performed in order to evaluate EGCG effect on pain perception. Mechanical allodynia (Figure 1A) and thermal hyperalgesia (Figure 2B) strongly increased already after two hours from surgical incision and lasted for five days gradually decreasing. EGCG administration already after 24 hours and three days increased the response to von Frey stimulation and heat source, reporting animals to pre-surgery levels at day four after surgery. To assess motor function, a rotarod test was performed (Figure 1C). Immediately after the surgery, animals showed impairments in motor coordination and took five days to recover. EGCG treatment significantly improved locomotor activity already after 24 h and three days from surgery.

### 2.2. Effect of EGCG Administration on WNT/FZ/β-Catenin Pathway Activation Induced by PO Pain 

**Figure 3:**
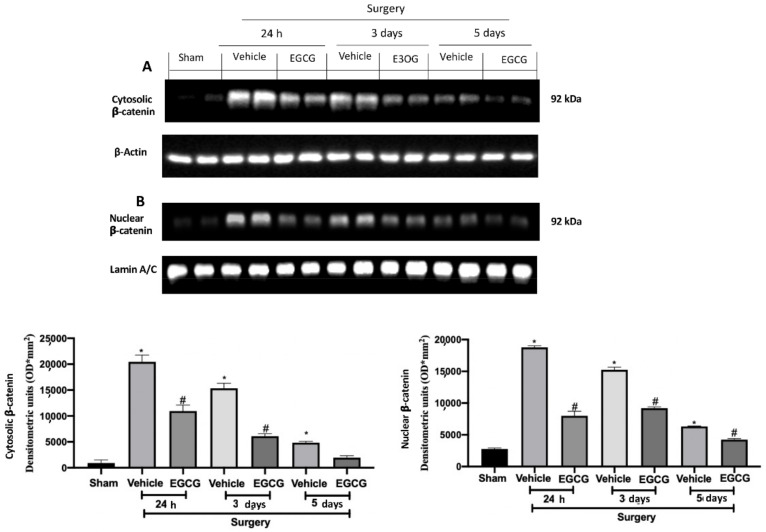
EGCG administration reduced β-catenin expression: Western blot analysis of: cytosolic β-catenin (**A**) and nuclear β-catenin (**B**) expression. A *p*-value of less than 0.05 was considered significant. * *p* < 0.05 vs. sham, # *p* < 0.05 vs. vehicle.

Western blot analysis showed increased WNT3a and FZ1 expression in samples harvested from vehicle treated rats 24 h and three days after surgery, as compared to sham animals, while no differences were detected at five days. EGCG administration significantly decreased WNT3a and FZ1 expression after 24 h and restored them to basal levels, three and five days after surgery (Figure 2A,B). FZ8 expression was significantly increased at all timepoints (24 h, and three and five days) as compared to the sham. EGCG treatment reduced FZ8 expression and restored it to basal levels after five days from surgery (Figure 2C). Additionally, in both cytosolic (Figure 3A) and nuclear (Figure 3B) fractions, β-catenin expression was significantly increased. EGCG administration strongly reduced β-catenin expression in cytoplasm and nucleus at all timepoints. 

### 2.3. Effect of EGCG Administration on GFAP and Iba-1 Activation Induced by PO Pain 

**Figure 4:**
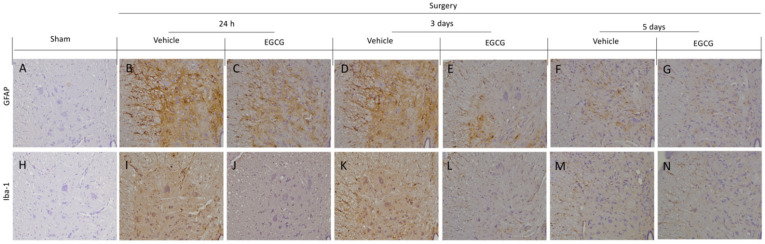
EGCG administration reduced GFAP and Iba-1 activation: Immunohistochemical analysis of GFAP: sham (**A**), vehicle 24 h (**B**), EGCG 24 h (**C**), vehicle three days (**D**), EGCG three days (**E**), vehicle five days (**F**), EGCG five days (**G**). Immunohistochemical analysis of Iba-1: sham (**H**), vehicle 24 h (**I**), EGCG 24 h (**J**), vehicle three days (**K**), EGCG three days (**L**), vehicle five days (**M**), EGCG five days (**N**). A *p*-value of less than 0.05 was considered significant.

Immunohistochemical analysis showed activated astrocytes and microglia were detected in vehicle treated rats. In particular, increased GFAP expression in vehicle treated rats at 24 h (Figure 4B) and three days (Figure 4D) after surgery, as compared to sham rats (Figure 4A), while, at five days, small levels of activation was detected (Figure 4F). EGCG administration significantly decreased GFAP expression at all timepoints (Figure 4C,E,G). Moreover, increased Iba-1 expression was found after 24 h (Figure 4I) and three days (Figure 4K), as compared to the sham animals (Figure 4H). Five days after surgery, Iba-1 expression slightly decreased in vehicle group (Figure 4M). EGCG treatment reduced microglia activation 24 h (Figure 4J), and three (Figure 4L) and five (Figure 4N) days after surgery.

### 2.4. Effect of EGCG Administration on pNR2B, pPKCγ, and pCREB Expression Induced by PO Pain

**Figure 5:**
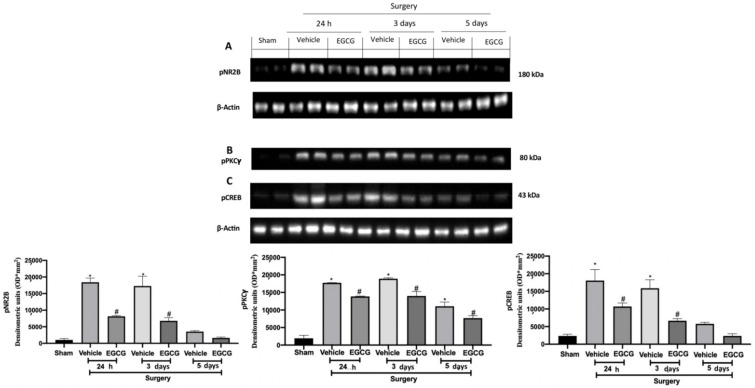
EGCG administration reduced pNR2B, pPKCγand pCREB expression: Western blot analysis of: pNR2B (**A**), pPKCγ (**B**), pCREB (**C**) expression. A *p*-value of less than 0.05 was considered significant. * *p* < 0.05 vs. sham, # *p* < 0.05 vs. vehicle.

Western blot analysis showed increased pNR2B expression (Figure 5A), pPKCγ (Figure 5B) and pCREB (Figure 5C) expression in samples harvested from vehicle treated rats 24 h and three days after surgery, as compared to sham animals, while at five days it slightly decreased. EGCG administration significantly decreased pNR2B (Figure 5A), pPKCγ (Figure 5B) and pCREB (Figure 5C) expression after at all timepoints.

### 2.5. Effect of EGCG Administration on Cytokines and NFkB Pathway Activation Induced by PO Pain

**Figure 6:**
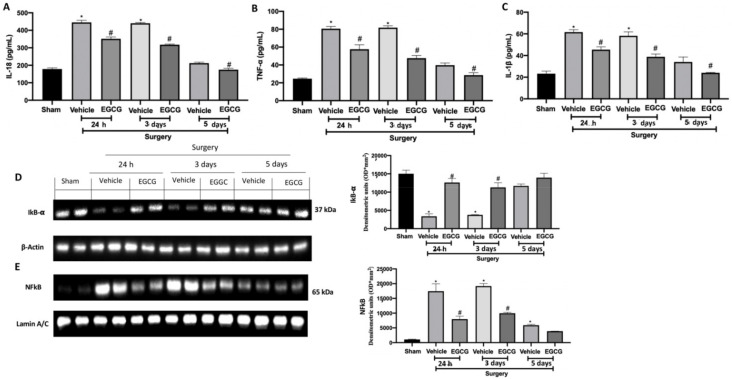
EGCG administration reduced cytokines and NFkB pathway activation: IL-18 (**A**), TNF-α (**B**), and IL-1β (**C**), Western blot analysis of: IkB-α (**D**) and NFkB (**E**) expression. A *p*-value of less than 0.05 was considered significant. * *p* < 0.05 vs. sham, # *p* < 0.05 vs. vehicle.

Increased levels of IL-18 (Figure 6A), TNF-α (Figure 6B) and IL-1β (Figure 6C) were detected in the vehicle group 24 h, and three and five days from surgery. EGCG treatment strongly reduced their expression, restoring it to basal levels at five days. Western blot analysis showed reduced IkB-α expression in the cytosolic fraction of the samples harvested from the vehicle group at all timepoints, as compared to the sham (Figure 6D). EGCG administration increased cytosolic IkB-α expression at 24 h and three days from surgery and restored it to basal levels after five. In the nuclear fraction, NFkB expression was increased in the vehicle group at all timepoints, as compared to the sham animals (Figure 6E). EGCG treatment significantly reduced nuclear NFkB expression at 24 h, and three and five days from surgery. 

### 2.6. Effect of EGCG Administration on iNOS, COX-2 and PGE2 Expression Induced by PO Pain

**Figure 7:**
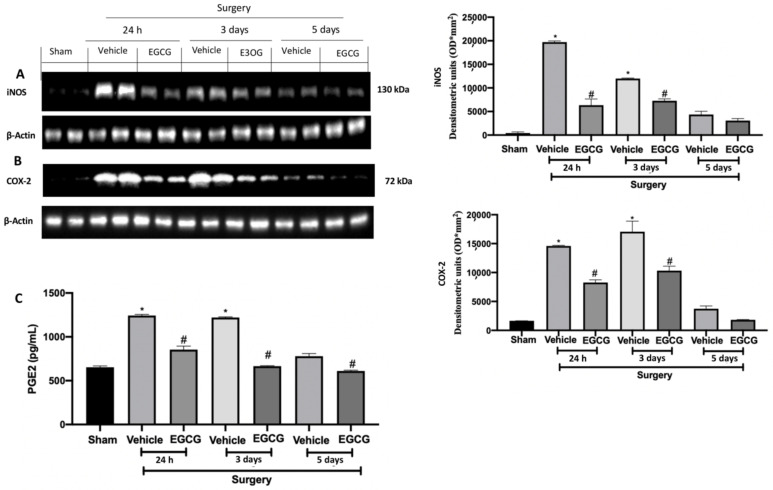
EGCG administration reduced iNOS, COX-2, and PGE2 expression: Western blot analysis of: iNOS (**A**), COX-2 (**B**) expression, PGE2 levels (**C**). A *p*-value of less than 0.05 was considered significant. * *p* < 0.05 vs. sham, # *p* < 0.05 vs. vehicle.

Western blot analysis showed increased iNOS and COX-2 expressions in tissues from vehicle treated rats 24 h, and three and five days after surgery, as compared to sham animals. EGCG administration reduced both expressions at all timepoints (Figure 7A,B). Additionally, PGE2 levels were significantly increased 24 h, and three and five days after surgery and EGCG administration reduced its levels (Figure 7C). 

## 3. Discussion

Patients consider PO pain to be one of the most unpleasant forms of surgical pain. Notwithstanding the increasing understanding of the molecular biology and neurology of PO pain, we still need to develop successful analgesics for pain control, including postoperative pain [32]. Thus, a significant number of patients continue to experience pain after surgery, which is not properly managed [33,34]. The surgical incision activates many molecular mechanisms that trigger and spread inflammation, oxidative stress and pain at peripheral and central levels [7,35]. This study was aimed to evaluate the mechanism of EGCG effect on PO pain in rats at different timepoints. EGCG showed important anti-inflammatory effects. In particular, mechanical hyperalgesia and thermal allodynia induced by the incision lasted for several days, while EGCG administration significantly alleviated hyperalgesia and allodynia. This antinociceptive would be ascribed to different molecular events induced by EGCG. We decided to evaluate the molecular mechanism activated by EGCG after 24 h, and three and five days from surgery. 

In particular, we investigated its effects on the WNT signaling pathway, evaluating the expression on WNT proteins. The β-catenin pathway is one of the most well studied in the propagation of pain and related injury and regeneration of the nervous system [15,19,20]. Among the 19 members of the WNT proteins family, WNT3a is the well-studied activator of this pathway [36,37]. Surgical incision induced an increase in the expression of WNT3a in the spinal cord. In particular, WNT3a expression was increased 24 h and three days from surgery, while after five days its expression partially decreased. 

WNT3a binding to the FZ receptors induced the activation of the WNT/β-catenin signaling pathway in the postsynaptic neurons. EGCG reduced WNT3a expression and also managed the expression of the FZ receptors at all timepoints. In particular, EGCG decreased WNT3a expression already after 24 h and three days from the surgery. Among the FZ receptors the two most well studied in the pain perception are FZ1 and FZ8. They differ from each other for the contribution to pain production and persistence. In particular, FZ1 is rapidly but transiently increased after injury, while FZ8 over-expression lasted for longer, contributing to pain persistence [13]. EGCG administration reduced both FZ1 and FZ8 expression, reducing both pain production and persistence 24 h, and three and five days from surgery. Activation of WNT/β-catenin signaling induces the β-catenin accumulation into the cytoplasm and its translocation into the nucleus [19,21]. EGCG once reduced the WNT3a and its receptor over expression also reduced β-catenin accumulation into the cytoplasm and translocation into the nucleus. From the neurochemical point of view WNT/β-catenin signaling is responsible of the pain-induced activation of microglia and astrocytes cells and upregulated the expression of the NR2B receptor and the related Ca2+-dependent pathway in the dorsal horn of the spinal cord [11,13,38,39]. EGCG administration reduced pain perception as displayed by the behavioral analysis and acting on the WNT/β-catenin pathway, reduced GFAP and Iba-1 over-expression both at all timepoints. Moreover, EGCG reduced the activation of the NMDA receptor subtype NR2B and the subsequent Ca^2+^—dependent signals Ppkcγ and cAMP response element-binding protein (CREB) in the spinal cord at all timepoints. PO pain has also an important inflammatory component [11,40]. WNT/β-catenin signaling regulates the IL-18 and TNF-α levels in the spinal cord that directly contribute to pain production and persistence. The increased expression of the pro-inflammatory cytokines is accompanied by elevation of IL-1β and NFkB pathway [41]. EGCG showed anti-inflammatory activity by reducing NFkB translocation into the nucleus and pro-inflammatory mediator over-expressions at all timepoints. NFkB manages the expression of several proteins such as COX-2 and iNOS [42]. COX-2, in turn, is major regulator of the increase in the PGE2 levels, which increases nociception following peripheral inflammatory stimuli. EGCG administration reduced COX-2 and iNOS expression and PGE2 levels 24 h, and three and five days from surgery. 

These results showed the important role of EGCG in modulating the WNT/β-catenin signalling pathway suppressing the production and persistence of PO pain and the related neurochemical and inflammatory alterations. 

## 4. Materials and Methods

### 4.1. Animals

Male Sprague Dawley rats (Envigo, Milan, Italy) were employed. This study was approved by the University of Messina Review Board for the care of animals (D.Lgs 2014/26 and EU Directive 2010/63).

### 4.2. Surgical Procedures

PO pain were performed as described previously [11]. Briefly, rats were anesthetized and placed dorsally. A 1 cm incision was made in the plantar hind paw. The underlying muscle was raised and longitudinally incised, leaving the insertion and origin of the muscle intact. A 5-0 nylon suture was employed to close the skin. The control group was anesthetized but no incision was performed. 

### 4.3. Experimental Groups

Rats were randomly divided in groups:

Group 1: sham + vehicle: Rats were anesthetized but no incision was performed. Vehicle solution (distilled water) was administered one hour, six hours after anesthesia, and daily by gavage.

Group 2: sham + EGCG: Rats were anesthetized but no incision was performed. EGCG (25 mg/Kg) was administered one hour, six hours after anesthesia, and daily by gavage.

Group 3: PO pain + vehicle: Rats were subjected to surgical procedure and EGCG (25 mg/Kg) was administered one hour, six hours after surgery, and daily by gavage.

EGCG dose was chosen based on literature [43]. Behavioral analyses were performed daily and animals were sacrificed at 24 h, and three and five days after surgery. Lumbar spinal cords were harvested for molecular analyses.

### 4.4. Behavioural Analysis

#### 4.4.1. Mechanical Hyperalgesia

A von Frey filament test was employed to evaluate hypersensitivity to a mechanic stimuli. The apparatus consists in a plastic box placed on a metal mesh floor and a force transducer equipped with a plastic tip [44,45,46]. After 15 min of acclimation the transducer was applied to the plantar surface until a sharp lift was observed. The mechanical threshold was recorded.

#### 4.4.2. Thermal Hyperalgesia

A plantar test was employed to evaluate thermal hyperalgesia. The apparatus consists of plastic chambers and a mobile unit with a heat source [47,48]. After 15 min of habituation, the heat source was applied to the plantar surface and the paw withdrawal latency was recorded.

#### 4.4.3. Motor Coordination

A rotarod test was employed to evaluate motor coordination. The apparatus consists of a horizontally oriented rotating cylinder suspended above a cage floor [49]. A training session was performed 24 h before test. The test was performed starting from a constant speed of 25 rpm, then the rotation was increased linearly at 20 rpm. The latency(s) for the first fall over a 4 min period was recorded.

### 4.5. Immunohistochemical Analysis

Lumbar spinal cord samples were fixed and embedded in paraffin. Immunohistochemical analysis was conducted, as previously described [50]. Sections were incubated overnight with: anti-glial fibrillary acidic protein GFAP (sc-33673) or anti-iba-1 (sc-3272). All sections were washed with PBS and then treated, as previously reported [51,52]. Stained sections were observed using a Leica DM6 microscope (Leica Microsystems SpA, Milan, Italy) following a typical procedure [53]. 

### 4.6. Western Blot Analysis

Lumbar spinal cord tissues were homogenized and Western blots were performed as already described [54,55]. A specific primary antibody, such as WNT3a (sc-80457), anti-FZ1, anti-FZ8, anti–β-catenin, anti-active β-catenin, anti-pNR2B, anti- pPKCγ, anti-*p*-CREB, anti-NFkB (sc-8008), anti-NOS2 (sc-7271), or anti-COX-2 (sc-376861), was mixed in 5% *w*/*v* nonfat dried milk solution and was incubated at 4 °C overnight. Afterwards, blots were incubated with peroxidase-conjugated bovine antimouse IgG secondary antibody or peroxidase conjugated goat anti-rabbit IgG for 1 h at room temperature [56,57]. To verify the equal amounts of protein, membranes were also incubated with the antibody against β-actin or lamin A/C. Signals were detected with enhanced chemiluminescence detection system reagent (Super-SignalWest Pico Chemiluminescent Substrate, Pierce, Monza, Italy) [58,59,60]. The relative expression of the protein bands was quantified by densitometry with Bio-Rad ChemiDoc XRS software (Bio-Rad, Milan, Italy) and standardized to β-actin or lamin A/C levels. Images of blot signals were imported to analysis software (v2003, Image Quant TL, Amersham Biosciences, Freiburg, Germany) [59]. The western blots analyses are representative of 3 different gels made by dividing the number of samples obtained from animals for each experimental group in different days [61,62].

### 4.7. ELISA

Serum IL-18, TNF-*α*, IL-1*β* and PGE2 levels were measured by enzyme-linked immunosorbent assay (Cusabio Biotech), according to the manufacturer instructions in pg/mL [63,64].

### 4.8. Statistical Evaluation

All values in the figures and text are expressed as mean ± standard error of the mean (SEM) of N observations. For in vivo studies, N represents the number of animals studied. The results were examined by one- or two-way analysis of variance followed by a Bonferroni post-hoc test for multiple comparisons. All results were normally distributed. A *p*-value of less than 0.05 was considered significant. A *p*-value of less than 0.05 was considered significant. * *p* < 0.05 vs. sham, # *p* < 0.05 vs. vehicle.

## Data Availability

The data presented in this study are available on request from the corresponding author.
